# From Midwife to Lead Perinatal Practitioner: A Utopian Vision

**DOI:** 10.1111/birt.12913

**Published:** 2025-03-10

**Authors:** John Pendleton, Sally Pezaro

**Affiliations:** ^1^ Faculty of Health, Education & Society University of Northampton Northampton UK; ^2^ Honorary Research Fellow Coventry University Coventry UK; ^3^ Research Centre for Healthcare and Communities Coventry University Coventry UK; ^4^ The University of Notre Dame Australia

**Keywords:** autonomy, gender, midwife, perinatal, practitioner, utopia

## Abstract

The professional title “midwife” is predicated on the understanding that people who access their services have a normative relationship between their gender and assigned sex. As trans and non‐binary people increasingly require access to midwifery services, this paper proposes an alternative professional title that is inclusive and liberates midwives from continuously reinscribing the sex/gender binary in their nomenclature. We work with Levitas's *Utopia as Method* framework to propose the title of Lead Perinatal Practitioner. Working through the archaeological, ontological and architectural modes, we explain the rationale for each component part of the title. “Lead” foregrounds the profession's relationship with autonomy, which is considered foundational but threatened by encroaching medicalization. “Perinatal” encompasses not just the birthing person but also the neonate and the physiological process and timeframe encapsulating pregnancy and birth currently absent. “Practitioner” captures the reflexivity, skill, and active engagement already inscribed in allied healthcare professions that use this title. We argue that when combined, they signal a trailblazing contribution towards the eradication of gender inequalities in the reproductive arena by uncoupling the profession from patriarchal oppression inscribed in the sex/gender binary, which has hitherto been positioned as the sine qua non of midwifery.

## Graphical abstract

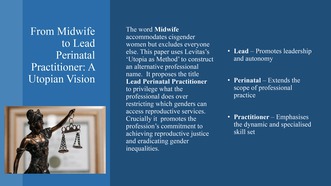

## 1 Introduction

Until recently, it has been unproblematically assumed that everyone who gives birth does so within a heterosexual relationship and experiences a normative relationship between their gender and assigned sex, particularly in the Global North. Accordingly, cisnormative, heteronormative, and heteropatriarchal services, administrative procedures, and structures have coalesced to both serve and ensure the perpetuation of the sex/gender binary in the perinatal space [1]. The sex/gender binary is a foundational element of what key theorists [2, 3] have described as invisible matrices existing to uphold patriarchy, a construct in which men (predominantly white) are privileged while inequalities and violence against women and minoritized groups are exacerbated. While neither sex nor gender is inevitably binary or automatically correlational [4, 5], under existing structures, the pregnant body reinscribes such binaries by being positioned as the apotheosis of femininity [6]. Moreover, reproduction remains inextricably linked to these imagined divisions [7], concepts that play a central role in perpetuating the subordination of all who give birth, the majority of whom will be cisgender women.

Within this binary understanding, midwives to date have played a crucial role both in safeguarding physiological birth from encroaching patriarchal techno‐medical advances and ensuring the psycho‐social safety of those they support. Central to this has been an idealized understanding that all midwives and birthing people should be ciswomen, interpellated through the word “midwife” which is commonly understood through semantic, philosophical, and etymological meanings as describing a cis woman who assists other cis women in childbearing [8]. This language reinscribes gender binaries and may be driven by biological determinism and essentialism in birth [9]. Nevertheless, the increasing visibility of trans and nonbinary people [10] accessing midwifery services disrupts these understandings. This forces midwifery to reassess its relationship with gender enshrined within its professional nomenclature. Moments of existential crisis and instability can be reconfigured as moments of great possibility. Just as theoretical lenses expose the conditions that have brought about the crisis, the possibilities it affords can also be glimpsed through using relevant frameworks. Inspired by Levitas's *Utopia as Method* framework [11], here we seek to imagine how changing midwives' professional title to disentangle itself from the sex/gender binary that has become the sine qua non of the profession to date might unlock new possibilities for the profession. We approach this task inspired by the work of Ahmed as “feminist killjoys” [12]. She invites us not to shy away from problematic issues simply because they might disrupt the normativity that exists to make others feel comfortable if doing so is important to extend freedoms to be more accommodating to everyone. We take as our starting point the word “midwife” arguing that it acts as a crucible for the crisis by upholding rigid understandings of sex and gender in childbearing. Indeed, language can be powerful in that it can either reinforce or destabilize cisheteronormative discourses [13]. Language can also act as a tool to interpret and construct gender [14]. It is also known to provoke powerful realizations and interpretations that speak to a future of new possibilities [15]. Considering the above, we argue that midwives, as experts in childbirth, are uniquely placed to deconstruct such harmful and oppressive patriarchal systems through language and lead trailblazing activity in the field of gender equity, synonymous with midwifery's long legacy rooted in social justice.

## 2 Utopia as Method

Utopia can be understood historically as a “non‐existent good place” [16] where all that is wrong can be corrected precisely because it is never subject to the unpredictability of existing political, economic, and social pressures. As Levitas [11] observes, this can lead to a tendency to present the status quo as a “smoothly operating, conflict free system” (*p*. 95) or which acknowledges imperfections and injustices as inevitable because an alternative is always aspirational but never achievable. To counteract this fatalism, Levitas builds upon the philosophical study of utopianism by offering a process of “speculative sociology” (*p*. 153) presenting three steps or modes in which to engage in and conceive of an imaginary reconstitution of society, a process which is “provisional, reflexive and dialogic” (*p*. 149). Making links between social theory, economic reality, and political processes, it is these steps we move through to reimagine wholly inclusive ways of being and doing outside the current status quo in midwifery. We begin by considering the first two modes—archaeological and ontological—in tandem to understand the problematic nature of the word “midwife”. We then move to the architectural mode to propose an alternative name for midwives and explain why each element of the term “Lead Perinatal Practitioner” can be configurative in order for the profession to stay relevant, offer a paradigm shift in reducing gender inequalities, and strengthen its status and reach.

## 3 Archaeological and Ontological Modes

Levitas [11] encourages us to consider the archaeological and ontological modes in dialogue with each other. The archaeological mode requires us to uncover an imaginary “good” society which has become obscured through sedimented layers of politics, policy, theory, and culture which all need to be re‐examined and reconfigured in order to construct it as it was originally desired in its purest form. The ontological mode asks what kinds of people and ways of being might be better than those which have come to uphold these imperfect systems. There is a dynamic interplay between these two modalities, no more so than in midwifery, which sees itself as both a way of *doing* and a way of *being* [17], the personal and the professional indivisible. We start from a position that midwifery has desired itself to be an ethically sound professional service as inscribed in key definitions given by the International Confederation of Midwives (ICM) [18, 19], grounded in feminist politics and principles to eradicate gender inequalities. Thus, in understanding what conditions are necessary for all individuals to flourish as midwives and service users, we use gender and feminism as the archaeological tools which allow us to excavate and critique that which has come to be understood as necessary for the functioning of the profession.

Feminism itself as a term is relatively recent, appearing in English for the first time in 1890 and remaining a pejorative and negative phrase to many until the 1960s [20] but continues to advance and expand to meet the needs of successive generations. From bell hooks understanding, feminism might mean “a movement to end sexism, sexist exploitation and oppression” [21]. In the context of perinatal services, midwifery is rooted in reproductive rights, the central site of birthing people's oppression. Levitas asks us to consider what is “valued, encouraged and genuinely enabled” (*p*. 153) in existing worlds and what might be possible in utopias. To do this, we must be willing to ask ethical questions about all taken‐for‐granted aspects of midwifery, the root of which is to consider the very word “midwife” itself and all that it contains. While the use of the term “midwife” was once understandable given earlier historical attempts to safeguard birth from patriarchal control [22], contemporary usage of the word “midwife” is problematic as described above. A move away from the use of sexed/gendered language which presently upholds patriarchy would enable a deconstruction of binary ideas in relation to childbearing [23]. The presence of gender diverse people accessing “maternity” services disrupts such norms, because it forces us to confront how words like “midwife,” “midwifery,” and “maternity” are excluding for example, of trans and non‐binary people. By identifying ourselves as “midwives,” we may be read as signaling our compliance with the status quo. However, utopian thinking allows us to recenter the problem away from bodies who access perinatal services to focus on challenging the patriarchal structures which cause harm. We can look away from who midwives are or care for and turn towards what midwives do. As midwives are not, nor should be “with women” exclusively, ultimately the word “midwife” fails to accurately communicate what the profession actually does or aspires to do. This is concerning as the professional identity of midwives is under threat [24, 25]. Thus, the professional title of “midwife” is arguably holding back progress by failing to command the same respect and value as other professions. This is perhaps because the word “midwife” is rooted in gendered and historical language rather than reflective of what the profession actually does, can and should do/be. While recognizing that the word “midwife” may conjure historical and societal images and feelings of nostalgia [26], it will be important to move past this and towards alternative language in pursuit of a strengthened professional identity and increased societal respect and value for the profession.

The word also reinforces oppressive colonialist ideals in relation to gender and birth, and in doing so hinders equality along with both reproductive [27] and gestational justice [28]. While we recognize that terminologies existed prior to European colonialism for those who supported and facilitated birth, the totalizing effect of colonialism has resulted in the word midwife becoming universally adopted in the Anglosphere. Indeed, the turn to decolonization is a good example of the power of engaging the archaeological and ontological modes to reimagine a better society and how precolonial terms can contribute to the ongoing debates we lay out in the architectural mode. Other parts of Europe have begun the process of uncoupling words for midwives from gender (verloskundige and maïeuticien [29] can replace more usual Dutch and French terms which translate as “wise woman”) and the impact of these changes is for future evaluation. Gender binaries have been socially and politically forced into being by colonial settlers to position white heteropatriarchy as the pinnacle of civilization [30]. This global dominance of cisheteronormativity and white supremacy as epistemes has led to gender being both racialized and sexualized at its foundations [31, 32]. Moreover, optimal perinatal care and curricula have also historically been viewed through a white western lens [33]. Thus, decolonization through deconstruction is required. Particularly as global majority women are almost four times more likely to die in the perinatal period than white women [34], a statistic which has remained largely static for over a decade.

Economically, childbearing is well recognized as reproductive or gestational labor driven by patriarchal capitalist ambition for profit [28]. Yet in pursuit of equality, this labor could usefully be untied from “women,” white repronormativity, and the gender binary [35]. By engaging in a dialogue between the archaeological and ontological modes, we have identified “doing” and “being” as concepts central to midwifery and foregrounded by a commitment to reproductive justice and gender equality which, we argue, can no longer be served by the gender‐exclusive terminology and which forecloses other possibilities. In moving away from the word “midwife,” we can unlink white cisheteronormative modes of femininity and reproduction from the socially perceived core aspects of being a woman. Ultimately, gender in relation to midwifery has thus far been understood in purely essentialist terms, is open to abuse, and coalesces in the words used to describe the profession, the professionals, and those who access it (e.g., “Midwives,” “Midwifery” & “Maternity”). We propose turning towards more progressive nomenclatures which promote justice and equality.

## 4 Architectural

The architectural mode demands that we are aspirational, looking forward to imagining what the future should look like and work backwards to see what resources are required to achieve it. In contrast to the rigidly gendered word “Midwife,” we propose a professional title which is both epicene and configurative in its meaning. The title “Lead Perinatal Practitioner” moves closer to capturing what midwives already do while maintaining a utopian vision. It leaves space to expand upon who midwives are, who midwives care for, and what midwives can—and should—do. Subsequently, we explain the meaning of the words both individually and collectively, pointing to the structures, systems and organizations which need to be either engaged with or altered in order to bring this new title into being, and in so doing, usher in new utopian opportunities for working and being inclusive in the service of justice.

### 4.1 Lead

Midwives already are leaders, and leadership is now enshrined in the United Kingdom (UK) in one of the five domains of the Nursing and Midwifery Council's (NMC) standards of proficiency for midwifery [36]. It is also contained in the word “autonomy” which is central to the ICM's definition of both the midwife as a person and a cardinal characteristic of midwifery as a profession [18, 19]. It is a word and a quality that has been used to distinguish the midwife from the nurse based on the history of the two professions, which have co‐existed in an “uneasy alliance,” [37] with midwifery frequently regarded as an extended role of nursing. It is important to acknowledge the historically symbiotic and porous relationship between the midwifery and nursing professions rather than pitting them against each other, given that both endure patriarchal subjugation by medicine [38]. Direct‐entry midwifery is associated with increased self‐reported confidence and competence in midwifery students, particularly important where the profession has the ability to make the greatest impact on reducing perinatal morbidity and mortality [39]. Establishing a new professional title for midwives could accelerate the process of uncoupling midwifery from nursing in terms of professional identity and regulatory bodies, strengthen the profession's valuable autonomy, and better clarify its scope of practice and accountability. It may also encourage the procurement of bespoke leadership development programs for midwives that are not subsumed under established programs centered upon nursing and other professional groups.

Autonomy is understood to incorporate the ability to make decisions independent of the control of others, to be self‐governing and self‐regulating both at an individual level and at a strategic level [40]. Midwives must use their autonomy to lead and coordinate perinatal care as the experts in pregnancy, childbirth, and newborn physiology. Nevertheless, globally, the ability for midwives to truly act autonomously is contested, with the increasing medicalization of birth identified as a factor in curtailing autonomous practice [41]. Midwives in New Zealand who practice within a continuity of care model experience greater levels of autonomy than those that can be afforded within obstetric‐led environments [42] which increasingly dominate in industrialized nations and account for rapidly escalating intervention rates and operative births [43]. The reasons for this are likely complex, multifactorial, and context‐specific, but what is known is that this medicalization curtails midwifery autonomy, deskills, and undermines midwives' confidence even in uncomplicated birth, and contributes to high attrition rates [44]. Ultimately, inscribing leadership in the professional title recenters the importance of autonomy within the profession.

### 4.2 Perinatal

The ICM's definition of midwives' scope of practice includes support, care, and advice throughout the childbearing continuum, conducting birth and newborn care, health promotion, and “women's health, sexual or reproductive health and childcare” [19] practicing across domestic and medical arenas. Within the State of the World's Midwifery Report 2021 [45], the scope of the midwife is broadened to include responding to violence against women and addressing sexual and reproductive rights, the provision of contraception, sexual health, and abortion care. The title of midwife, in contrast, is not broad enough in its most understood meaning of “with woman” to be able to encompass this wide professional remit nor the personhood of the newborn, families, and communities that wrap around the birthing parent. Rather than relying on a professional title that does not refer to the scope of professional practice, we propose “Perinatal” as a key word, “natal” focusing on the physiological process of pregnancy and birth as the site around which the professional builds their scope of practice. The word “natal,” still with its historic origins, also shifts and expands the focus from being with “woman” to encompass the childbearing person, the pregnancy, and the neonate.

Similarly, the period when the midwife is the lead healthcare professional is presently ill‐defined; the Lancet series on midwifery refers to the role of the midwife as providing care from pre‐pregnancy to the “early weeks of newborn infants’ life,” [46] while a dictionary definition of “perinatal” is simply the period around (Peri) the time of birth [47]. The term “Perinatal” is also used as a legal definition of time spanning from the 22nd week of pregnancy to 7–28 days post birth and in the UK the term has become closely associated with perinatal mental health, understood to refer to any time during pregnancy and the first year of life following birth [48]. Indeed, the way in which we define perinatal mortality also encompasses the year following childbirth [49]. The flexibility in determining what is meant by the term “Perinatal” works in synergy with the autonomy foregrounded in the word “Lead.” While regulatory bodies may be moving away from delineating when a midwife could or should be involved in care, local policies and protocols frequently limit this to the first few days or weeks after a baby is born [45], leading to fragmented care. The word “Perinatal” instead captures a timeframe determined by the holistic needs of the service user, and thus more usefully centers the relationship between professional and service user back to the professional title.

## 5 Practitioner

Meanings ascribed to the word “midwife” are multiple, unstable, and at times fiercely contested [8]. Current understandings of midwifery focus upon its ontological tradition of “masterly inactivity” [17] as a counterpoint to the hegemony of obstetric intervention, which has come to dominate models of facilitating childbirth in the Global North and rapidly industrializing countries [42]. We posit that the word “Practitioner” can bridge these two binaries, as it implies active engagement rather than a fixed end point that forecloses other possibilities. The word shifts the focus from “being” (with woman) which does not do justice to the dynamic skill set required of all professionals, to a more active role and the “doing” of care using these skills. “Practitioner” also implies a reflexive professional who is open to learning and constantly in pursuit of improvement for both the self and for those they care for. Moreover, it acknowledges the long training and high levels of skill and specialism required for a role such as this [50]. The term “Practitioner” has common currency within healthcare (e.g., General Practitioner, Operational Department Practitioner, Mental Health Practitioner) and within nursing disciplines can refer to a more experienced professional who has been given autonomy to diagnose and treat specific conditions without referral to a doctor, akin to the way midwives are already able to work at the point of registration.

## 6 Lead Perinatal Practitioner

The three words included in the proposed professional title of Lead Perinatal Practitioner are individually significant in describing and directing midwives' professional remit. They foreground autonomy, extend the remit of who is cared for and for how long, and emphasize the skill of midwives' clinical expertise. However, when combined they offer a powerful and emancipatory way forward because, significantly, they do not limit the personhood of who can be encompassed by this care, nor do they imply in their configuration who is welcome (or not) to join the profession. Considering the above, a move away from the title of “Midwife” to “Lead Perinatal Practitioner” would be trailblazing in reducing inequalities through the deconstruction of harmful patriarchal constructs and decolonization in‐keeping with the United Nations Millennium Development Goals for 2030 to achieve gender equality [51]. Of course, the midwifery profession would not be the first to adopt a gender‐neutral professional title, as other examples, some less oppressed by the patriarchy demonstrate (e.g., the “policeman” has become the “police officer,” the “fireman” has become the “firefighter”) but as the most highly gendered profession [52], it may achieve the highest impact in doing so.

This paper does not call for the eradication of the word “midwife” in a historical or nostalgic context. Indeed, we maintain the need for a usable past to shape our future [25]. Rather, we propose those who join the profession in future and those bodies and organizations currently key within it adopt the new utopian vision we have illustrated here. We can envision this by imagining for example the Royal College of Midwives becoming the Royal College of Lead Perinatal Practitioners, or the ICM becoming the International Confederation of Lead Perinatal Practitioners. The NMC may also reconfigure itself as a regulator better able to represent the profession. While we have focused upon a utopian vision progressing away from the word “midwife,” we recognize that usage of related etymology (e.g., midwifery/maternal/maternity) is also problematic. Consequently, future works could usefully apply this same method to arrive at a utopian vision for language associated with the profession as a whole.

## 7 Conclusion

In looking to the future, an alternative, inclusive, and accurate title such as “Lead Perinatal Practitioner” may afford the profession more authority, value, and professional identity where it is currently lacking. Seemingly, midwifery has been historically constrained thus far, in a nostalgic image of what and how it has been imagined and romanticized to be. While our aim is to achieve a paradigm shift in relation to midwives and midwifery, we still seek to learn and progress from our past, rather than erase it. Thus far, cisgender, heterosexual white women have been centered and privileged in both midwifery and childbearing. At the same time, justice remains unserved in the cisnormative and heteropatriarchal systems apparent in perinatal services which oppress us all, predominantly cisgender global majority women. We acknowledge that where people are used to privilege and being centered, moving toward equality can feel like oppression. There may also be discomfort and cognitive dissonance where there is a struggle to reconcile previously held beliefs in relation to gender, sex, and professional identities. This paper has shown the need for such change and, via Levitas, has provided a blueprint for proposing an alternative title which serves as the start of a respectful conversations with the midwifery community on how we wish to be named. If we are truly committed to working towards a future where gender in the reproductive arena is no longer synonymous with inequity, it is time to get uncomfortable.

## Conflicts of Interest

The authors declare no conflicts of interest.

## Data Availability

Data sharing not applicable to this article as no datasets were generated or analysed during the current study.
